# Changes in gene body methylation do not correlate with changes in gene expression in Anthozoa or Hexapoda

**DOI:** 10.1186/s12864-022-08474-z

**Published:** 2022-03-25

**Authors:** Groves Dixon, Mikhail Matz

**Affiliations:** Department of Integrative Biology, University of Texas at Austin, Austin, USA

**Keywords:** Epigenetics, DNA methylation, Gene regulation, Plasticity, Transcriptomics, Gene body methylation, Coral

## Abstract

**Background:**

As human activity alters the planet, there is a pressing need to understand how organisms adapt to environmental change. Of growing interest in this area is the role of epigenetic modifications, such as DNA methylation, in tailoring gene expression to fit novel conditions. Here, we reanalyzed nine invertebrate (Anthozoa and Hexapoda) datasets to validate a key prediction of this hypothesis: changes in DNA methylation in response to some condition correlate with changes in gene expression.

**Results:**

In accord with previous observations, baseline levels of gene body methylation (GBM) positively correlated with transcription, and negatively correlated with transcriptional variation between conditions. Correlations between changes in GBM and transcription, however, were negligible. There was also no consistent negative correlation between methylation and transcription at the level of gene body methylation class (either highly- or lowly-methylated), anticipated under the previously described “seesaw hypothesis”.

**Conclusion:**

Our results do not support the direct involvement of GBM in regulating dynamic transcriptional responses in invertebrates. If changes in DNA methylation regulate invertebrate transcription, the mechanism must involve additional factors or regulatory influences.

**Supplementary Information:**

The online version contains supplementary material available at 10.1186/s12864-022-08474-z.

## Background

The regulatory function of DNA methylation in invertebrates, if any, remains unclear. This contrasts with DNA methylation in mammals, where its role in transcriptional repression is well established. Mammals exhibit high genomic DNA methylation levels, with 70-80% of CpGs methylated [1]. This is hypothesized to have evolved for genomic defense, with methylation of transposons’ promoter elements silencing their expression [2]. Promoter methylation is also linked with mitotically heritable silencing of some mammalian genes, such as X-inactivated, imprinted-, and germline-specific genes [3]. Mitotically heritable methylation patterns are enabled by the maintenance methyltransferase DNMT1, which is homologous in vertebrates and invertebrates [4]. Methylation of gene bodies (gene body methylation; GBM), in contrast, is associated with actively transcribed genes [5]. Regions of accessible chromatin also tend to be weakly methylated or unmethylated [3], consistent with an influence of methylation on transcription factor binding [6].

In invertebrates, DNA methylation occurs predominantly in the form of GBM [7] and is positively correlated with expression [8]. Despite its association with active expression, there is little evidence that invertebrate methylation directly regulates transcription. For instance, several studies failed to detect substantial differences in GBM across invertebrate cell types or developmental stages [9–11] despite profound transcriptome differences (although see Liew et al. [12]). Conversely, removal of GBM by knockdown of DNMT1 enzyme did not significantly alter gene expression in a milkweed bug [13]. Similar results have been observed in plants [14–16]. Together, these studies indicate that changes in GBM are neither necessary nor sufficient to induce changes in transcription.

As invertebrate coding genes are separated into highly methylated and lowly methylated classes, we earlier hypothesized that methylation class serves as a regulatory signal and that the highly and lowly methylated classes of genes undergo group-level changes in methylation and transcription. In a previous study of *A. millepora*, we observed reciprocal changes in GBM and transcription depending on methylation class [17]. The study used MBD-seq and Tag-seq to examine changes in GBM and transcription in colonies of *A. millepora* reciprocally transplanted between two environments. In colony fragments transplanted to the environmentally favorable location, the highly methylated class of genes decreased in methylation, while the lowly methylated class increased. Looking at transcription in these colony fragments, the highly methylated class tended to be upregulated upon transplantation, while the lowly methylated class tended to be downregulated. The inverse pattern was observed in an independent set of coral samples transplanted from the more favorable to the less favorable location [17]. Based on this observation, we proposed a regulatory mechanism in which opposing class-level changes in GBM produce reciprocal class-level changes in transcription. As environmentally responsive genes tend to be in the lowly methylated class, and housekeeping genes tend to be in the highly methylated class, this mechanism could allow broad shifts between responsive, ‘problem solving’ transcriptional profiles and homeostatic house-keeping profiles. As the hypothesis involved reciprocal shifts between the two methylation classes, we refer to it as the ‘seesaw hypothesis’.

**Fig. 1:**
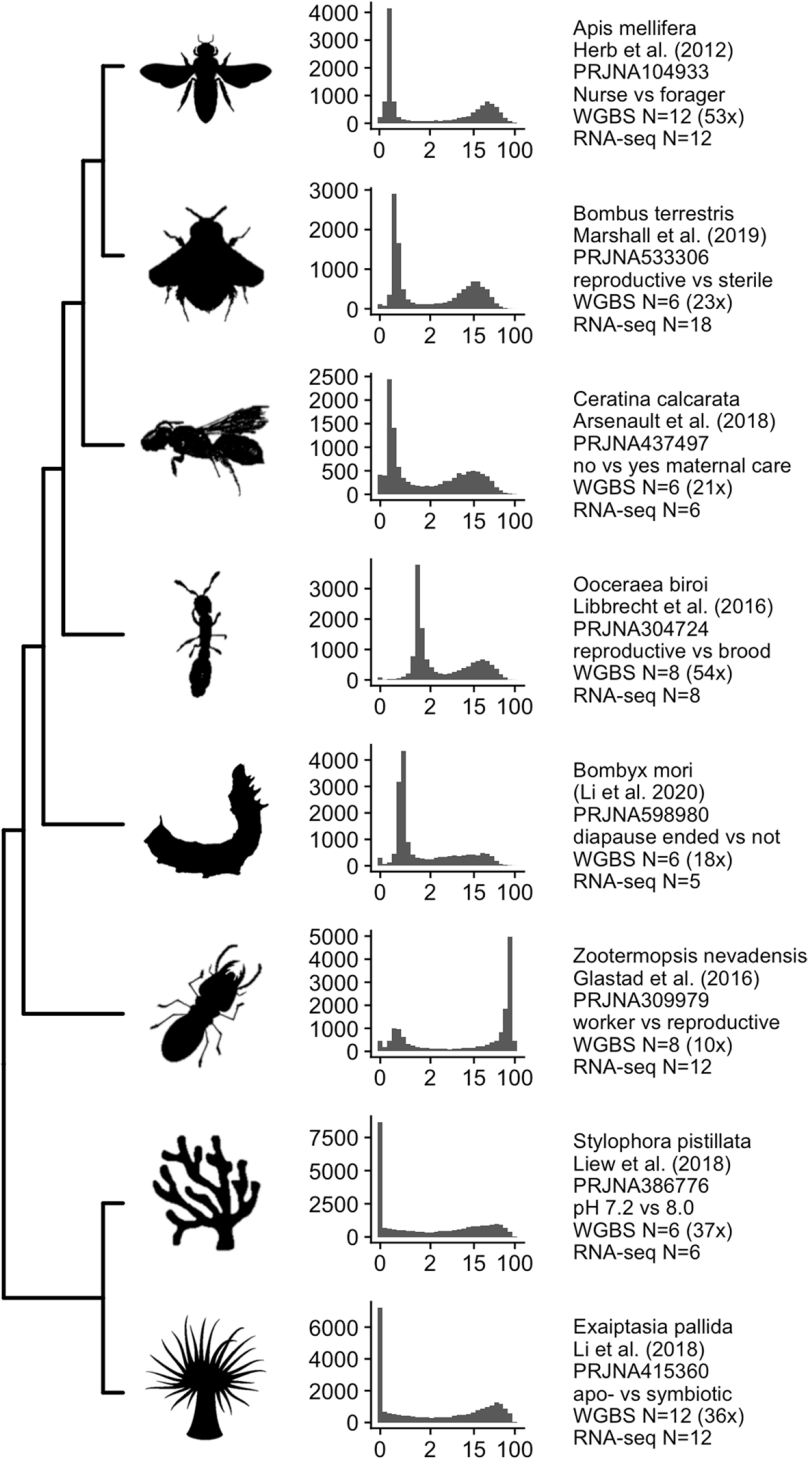
Overview of species covered by previously published datasets and the distribution of GBM levels in each. X-axes for histograms show percent methylation (summed across all CpG sites within the gene and averaged across all samples from each study) on the log_2_ scale. The Y-axes show the counts of genes. Text reports the species, reference, NCBI Bioproject accession, the treatment groups compared here, the Whole Genome Bisulfite sample size (with mean genomic coverage), and the RNA-seq sample size for each study

Here, we re-analyze publicly available methylomic and RNA-seq data from three Anthozoa and six Hexapoda studies (Fig. 1) to evaluate relationships between invertebrate DNA methylation and transcription. For each study, we contrast methylation- and transcriptional differences between two conditions. The Anthozoan studies contrasted polyp types in the coral *Acropora millepora* [18], pH treatments in the coral *Stylophora pistillata* [12], and symbiotic state in the sea anemone *Exaiptasia pallida* [19]. Hexapoda studies included different reproductive states in ants (*Ooceraea biroi*) [20], bumblebees (*Bombus terrestris*) [21], and termites (*Zootermopsis nevadensis*) [22], different subcastes in honeybee (*Apis mellifera*) [23], differences in maternal care in carpenter bee [24], and different diapause states in silkworm (*Bombyx mori*) [25]. Using these diverse datasets, we first confirm previous findings that baseline GBM levels are bimodally distributed across coding genes, are positively associated with baseline transcription level, and are negatively associated with transcriptional variation. We next examine the hypotheses that changes in GBM and/or promoter methylation between conditions correlate with changes in transcription. Lastly, we assess three components of the seesaw hypothesis of Dixon et al. (2018, 17]: (1) the highly- and lowly-methylated gene classes undergo reciprocal changes in GBM, (2) the two classes undergo reciprocal changes in transcription, (3) class-level changes in transcription will be in the opposite direction of class-level changes in GBM.

## Results and discussion

### Confirming previous relationships between GBM and transcription

**Fig. 2:**
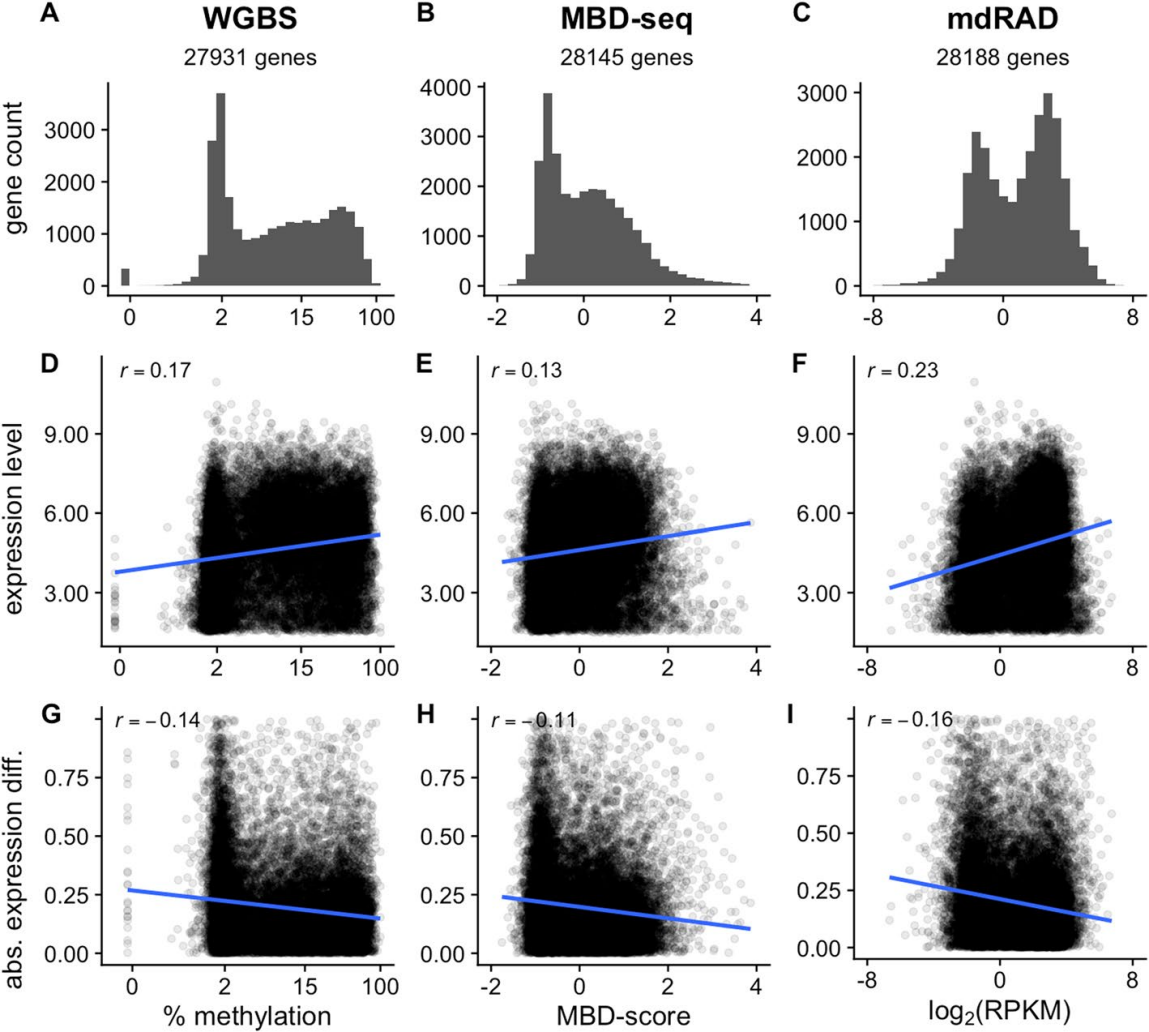
Associations between GBM level and gene expression patterns in *A. millepora* based on three different methylation assays. **A**-**C** Distribution of GBM levels **D**-**F** Relationship between GBM level and mRNA abundance **G**-**I** Relationship between GBM level and the absolute value of differential mRNA expression between axial and radial polyps. X-axes for the assays are as follows. Whole Genome Bisulfite Sequencing (WGBS): percent methylation (summed across all CpG sites of each gene and averaged across all samples) on the log_2_ scale; Methylation Binding Domain Sequencing (MBD-seq): the log_2_ difference in fold coverage between the captured and unbound fractions (MBD-score, see methods); methylation dependent RAD-seq (mdRAD): Reads per Kilobase of gene sequence per Million reads on the log_2_ scale (log_2_(RPKM)). The correlation coefficient is given in the upper left hand of each plot

We first sought to corroborate previous findings on the distribution of GBM, and its relationship to gene expression patterns. Using three different methylation assays, we confirmed that GBM in *A. millepora* shows a characteristic bimodal distribution, separating genes into highly methylated and lowly methylated classes (Fig. 2 A-C). We then confirmed that GBM level is associated with average mRNA abundance (Fig. 2 D-F), and negatively associated with differential expression between polyp types (Fig. 2 G-I). Hence, regardless of the method used to measure methylation, GBM shows the expected distribution and associations with gene expression in *A. millepora*.

**Fig. 3:**
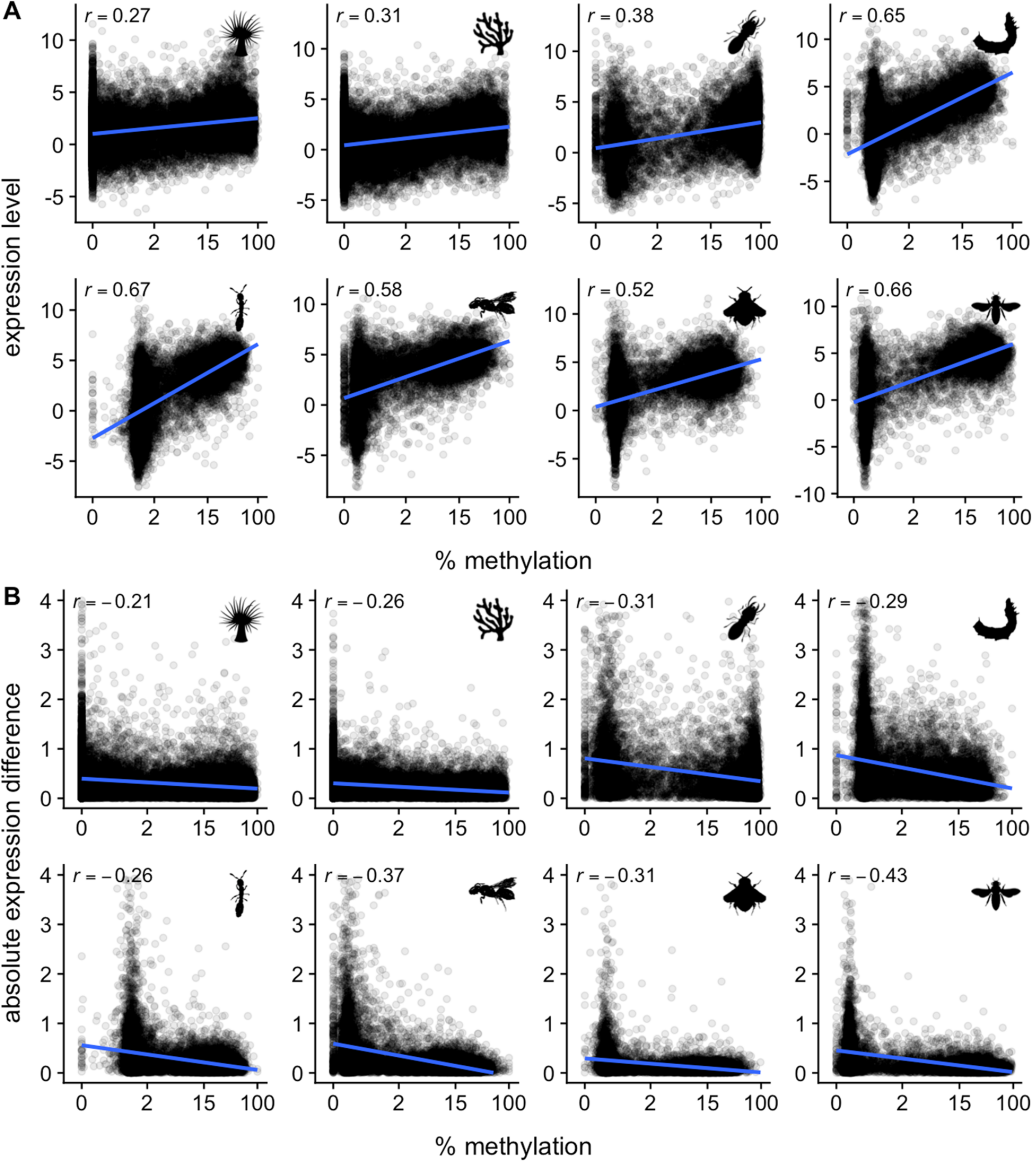
Associations between GBM level and gene expression patterns. **A** Relationship between GBM level and mRNA abundance (RNA-seq RPKM). **B** Relationship between GBM level and the absolute value of expression differences between phenotypic groups (contrasts given in Fig. 1). X-axis shows percent methylation on the log_2_ scale. The correlation coefficient is given in the upper left of each plot

The other studies showed similar results. While the relative sizes and means of the peaks varied by dataset, these were also bimodally distributed (Fig. 1) and similarly associated with mRNA expression patterns (Fig. 3). The coefficient of variation (standard deviation (RPKM) / mean(RPKM) computed from control replicates) was similarly negatively related to GBM (Fig. S1). Relationships between GBM and expression level were stronger among the insects than the cnidarians. Hence, GBM was positively linked with transcription level and negatively linked with transcriptional variation in all the studies included here.

### No correlation between changes in GBM and changes in transcription across genes

**Fig. 4:**
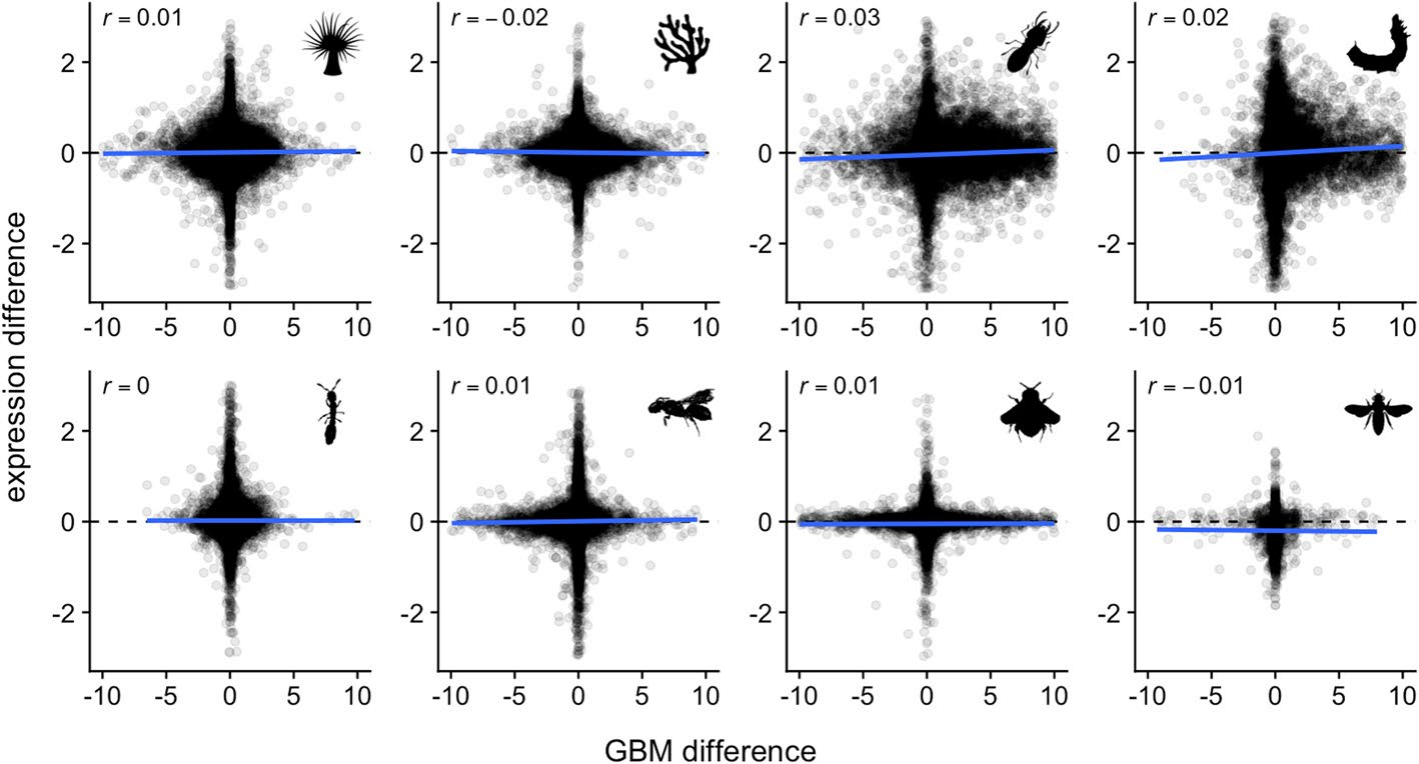
Changes in GBM and transcription between phenotypic conditions showed no clear relationship. X axes indicate differential GBM estimated using MethylKit. Y axes indicate differential transcription estimated using DESeq2. Both axes are on the log_2_ scale. Correlation coefficient is given in the upper left of each plot. Contrasts used to compute differential GBM and transcription are given in Fig. 1

As GBM is associated with elevated expression, a simple hypothesis is that increasing GBM increases transcription. Our re-analysis of GBM- and mRNA differences between phenotypic groups shows that this is not the case. Using three different methylation datasets in *A. millepora*, we found that measurements of GBM differences between polyp types showed no consistent association with transcriptional differences (Fig. S2). This was also the case for each of the other datasets (Fig. 4). Repeating this analysis using only differentially expressed genes (DESeq2 FDR < 0.1) or differentially methylated genes (MethylKit FDR < 0.1) produced similar results, with no clear association between differential GBM and differential transcription (Fig. S3). Hence, in Anthozoa and Hexapoda, GBM and transcription show no linear covariation between phenotypic conditions.

### No correlation between changes in promoter methylation and changes in transcription

As promoter methylation is associated with gene silencing in vertebrates, we tested whether changes in promoter methylation correlate with changes in gene expression in invertebrates. Specifically, we tested whether differences in methylation in windows 1Kb upstream of genes between phenotypic conditions predicted differences in mRNA levels. As with GBM, we found no reproducible relationship between changes in promoter methylation and changes in expression for *A. millepora* (Fig. S4), or any of the other studies (Fig. S5).

### Class-level shifts in GBM and transcription

**Fig. 5:**
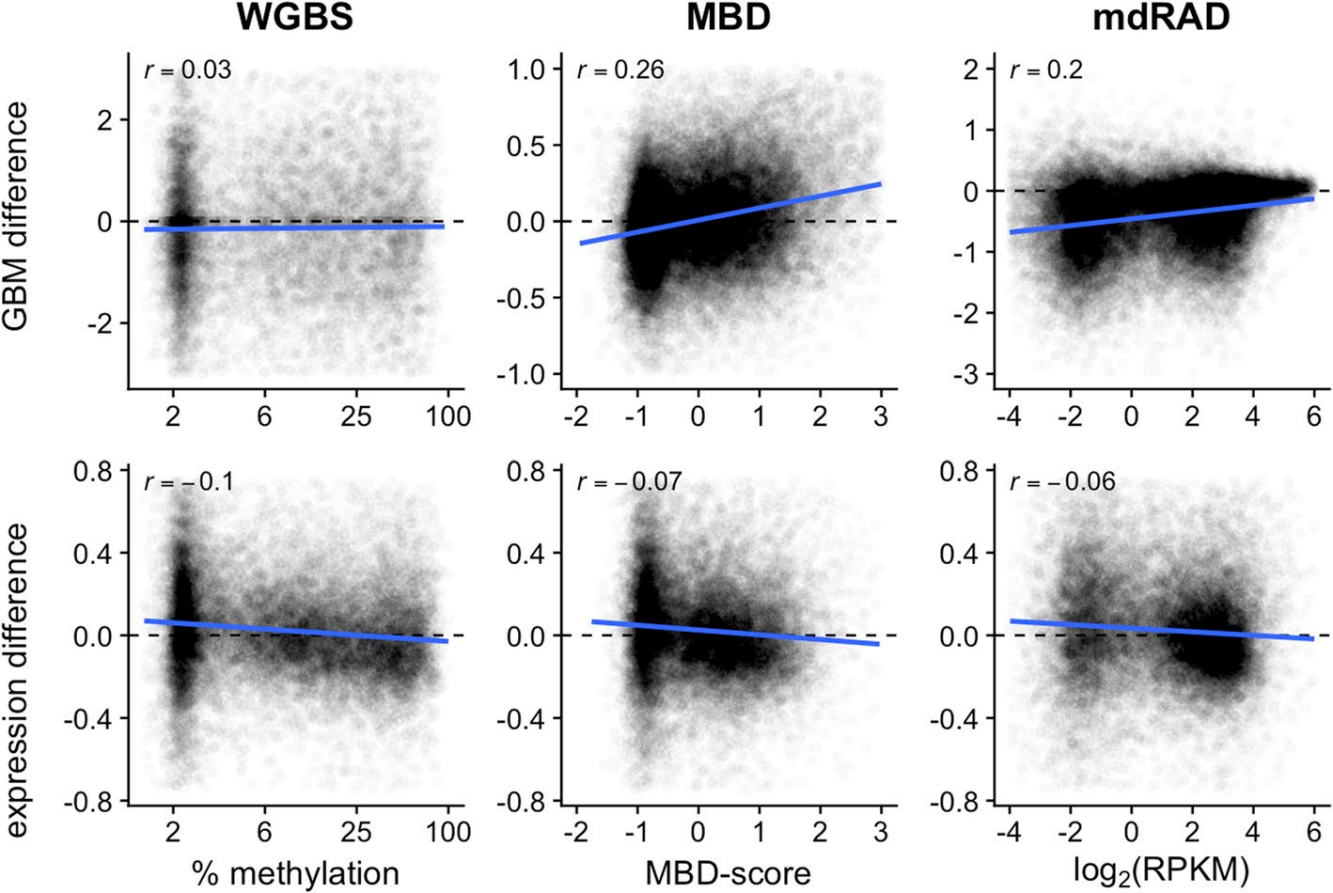
Class-level shifts in GBM and transcription in *A. millepora*. X axes show baseline GBM level averaged across all samples. Y axes shows differential GBM between polyp types (top panels) and differential transcription between polyp types (bottom panels). Top panels: Differential GBM between polyp types was linked with baseline GBM level for the MBD-seq and mdRAD datasets, but not for WGBS. Bottom panels: For all three methylation datasets, the low GBM class of genes was somewhat upregulated in axial compared to radial polyps, and the high GBM class was somewhat downregulated

Regarding the seesaw hypothesis, results from the *A. millepora* dataset were inconclusive. The expected seesaw pattern was observed based on MBD-seq and mdRAD in axial compared to radial polyps: the highly methylated class increased in methylation level while the lowly methylated class decreased in methylation. However, this pattern was not apparent in the WGBS dataset (Fig. 5). The reason for the match between two methods but not the third one is unclear. While there might be a technical issue with this specific WGBS dataset, it is concerning that of the three GBM-detection methods the only one that failed to show the seesaw pattern is the one that is supposed to be the most reliable [26]. Looking at the transcriptional data, based on all three methylation assays, the lowly methylated class was somewhat upregulated, and the highly methylated class was somewhat downregulated (Fig. 5). While this was consistent with our previous observations [17], the overall weakness of these effects plus the disagreement with the WGBS data does not allow claiming confident support for the seesaw hypothesis.

**Fig. 6:**
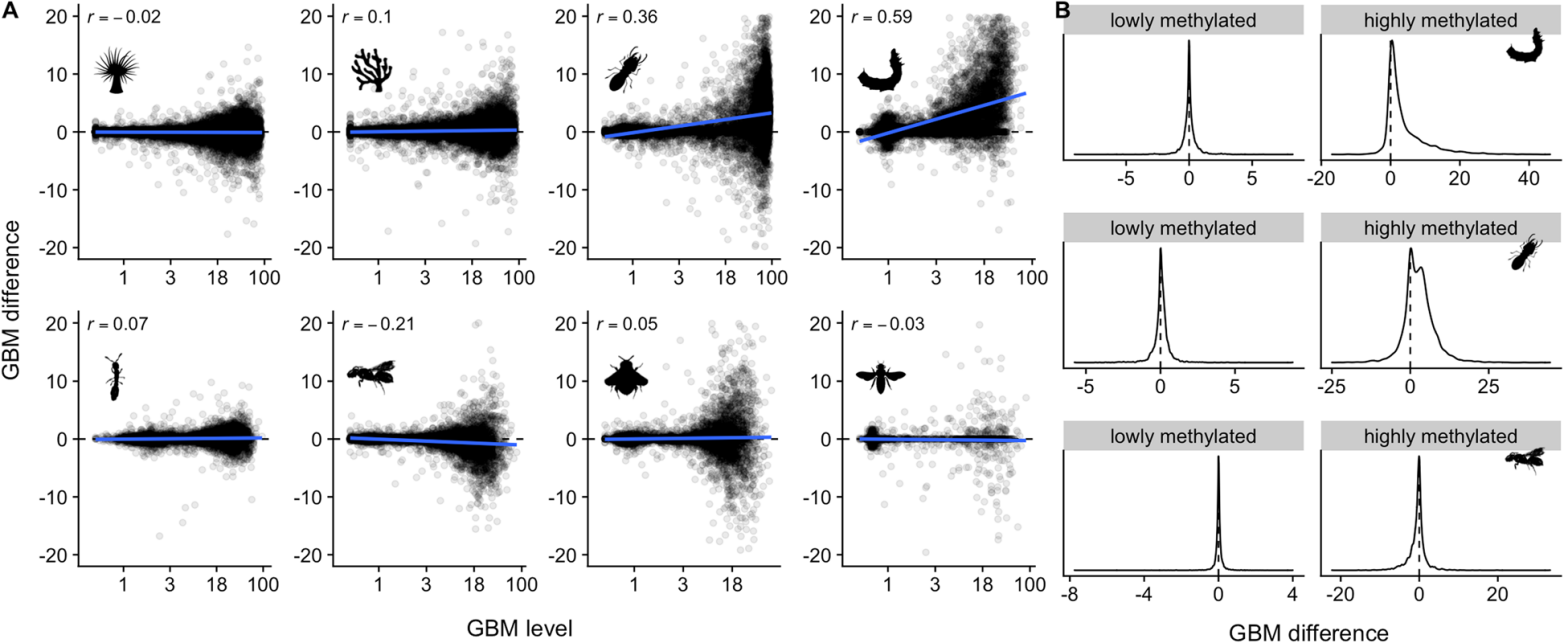
Class-level shifts in GBM. **A** Scatterplots of GBM change against mean GBM level. Only three studies (Silkworm, Termite, and Carpenter bee) showed group-level changes in methylation between conditions. **B** Density plots of GBM change for Silkworm, Termite, and Carpenter bee. Average shifts were observed in the highly methylated class, but not in the lowly methylated class

Among other studies, we never observed coordinated class-level changes of GBM and transcription. There were three cases (silkworm, termite, and carpenter bee) of class-level shifts in methylation, but unlike Dixon et al. (2018) [17] only the highly methylated class changed (Fig. 6). The silkworm dataset shows this most clearly, with a strong average increase in methylation level for the highly methylated class, but little or no average change in the lowly methylated class (Fig. 6). While the methylation level measurements of the lowly methylated class ranged from roughly 0 to 3% across the datasets, it seems likely that GBM in this class is essentially negligible, and does not change.

**Fig. 7:**
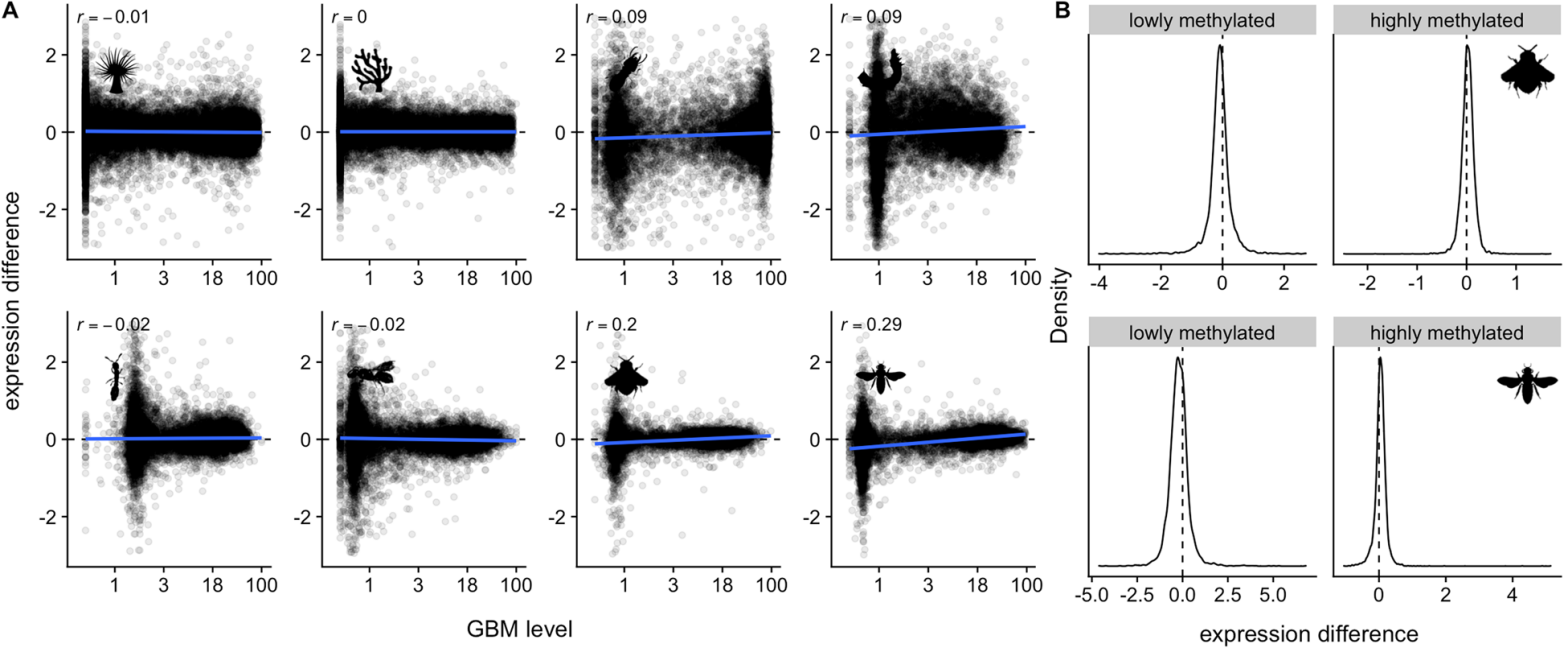
Two cases of inverse GBM class-level shifts in transcription. **A** Transcriptional differences (log_2_ fold change) plotted against GBM level (% methylation). **B** Density plots of the log_2_ fold changes in transcription for the lowly methylated and highly methylated classes for Bumblebee (contrasting reproductive vs sterile individuals) and Honeybee (contrasting nurse vs forager subcastes). In these two cases, the lowly methylated class tended to be downregulated and the highly methylated class tended to be upregulated

In contrast to Dixon et al. (2018) [17], class-level changes in methylation in non-*Acropora* studies were not associated with class-level changes in transcription (Fig. 7). Two of the eight studies did however, demonstrate class-level changes in transcription alone. In these two cases (honeybee and bumblebee), the lowly methylated class was downregulated on average, and the highly methylated class upregulated on average (Fig. 7). In summary, while some aspects of the “seesaw” hypothesis proposed by Dixon et al. (2018) [17] were detected in several cases, the hypothesis was not fully supported by any of the studies included here. We conclude that, whenever observed, GBM and gene expression seesaw patterns are unlikely to be directly functionally related. More likely, they reflect some other processes influencing the bulk of gene regulatory states, for example differences in cellular proliferation or growth.

A possibility that can rescue the regulatory role of invertebrate DNA methylation is that it interacts with other epigenetic modifications, which must be included to accurately model invertebrate gene expression [27]. For instance, in vertebrates, methylation of regulatory elements is known to influence the binding of transcription factors and their gene regulatory effects [28–30]. Regulatory effects of methylation could be further influenced by interactions with other chromatin features such as histone modifications [31–33]. As differential GBM has little or no power in predicting differential transcription between invertebrate phenotypic states, uncovering regulatory functions of DNA methylation in invertebrates will likely require interrogation of such additional features.

## Conclusions

Here we used published methylomic and transcriptomic data from Anthozoa and Hexapoda to examine how DNA methylation relates to transcriptional variation between different phenotypic conditions. We found that, as previously reported, GBM is bimodally distributed, and that higher GBM levels are associated with elevated transcription and less transcriptional variation. However, differences in GBM between conditions showed no consistent linear association with differences in transcription. As there were often detectable differences in both GBM and transcription (Fig. S6), this indicates that changes in GBM are neither necessary nor sufficient to induce changes in transcription in invertebrates. Methylation differences 1 Kb upstream of the first exon also showed no association with differences in transcription. In conclusion, if shifting methylation patterns regulate invertebrate transcription, the mechanism is more complex than can be captured by a simple linear relationship between these two variables.

## Methods

### Previously published datasets

Previously published WGBS and RNA-seq datasets from invertebrate species are shown in Fig. 1. The criteria for selecting these projects were: 1) the project focused on an invertebrate species 2) the project included at least two conditions, such as environmental exposure, or caste. 3) the project characterized DNA methylation using Whole Genome Bisulfite Sequencing (WGBS) 4) the project characterized transcription using RNA-seq 5) reads were available on the NCBI SRA database. Experimental methods from some projects allowed for multiple comparisons, however for simplicity, we focused on contrasts that seemed likely to induce the greatest epigenetic change. The comparisons we made are as follows. For the anemone *Exaiptasia pallida* [19], we compared aposymbiotic (*N* = 6) to symbiotic (*N* = 6) individuals. For the smooth cauliflower coral *Stylophora pistillata* [12], we compared only the most extreme pH treatment (pH 7.2; *N* = 3) to controls (pH 8.0; *N* = 3). For silkworm *Bombyx mori* [25], we compared diapause terminated (*N* = 3) to diapause destined (*N* = 3) eggs. For the termite *Zootermopsis nevadensis nuttingi* [22], we compared winged reproductive alates of both sexes (*N* = 4) to larval instars (workers) of both sexes (*N* = 4). For the small carpenter bee *Ceratina calcarata* [24] we compared newly eclosed adults that developed without maternal care (*N* = 3) to those that received maternal care (*N* = 3). For bumblebee *Bombus terrestris* [21], we compared reproductive (*N* = 3) to sterile castes (*N* = 3). For honeybee *Apis mellifera* [23], we compared nurse subcastes (*N* = 6) to worker subcastes (*N* = 6). For the clonal raider ant *Ooceraea biroi* [20], we compared individuals in the reproductive phase (*N* = 4) to those in brood care phase (*N* = 4). For *A. millepora*, DNA methylation was measured using three assays, WGBS, MBD-seq, and a variation of the methylRAD assay (described by Wang et al. 2015 [34]) called mdRAD [18], and transcription was measured using Tag-based RNAseq [35]. Here we compared tissue from axial polyps (taken from the very tips of branches) to radial polyps (taken from the sides of branches). The presence of the maintenance methyltransferase DNMT1 was confirmed in each of these species by blasting the human protein sequence against each of their reference proteomes, each with an e-value of 0.

### WGBS data processing

Raw reads were trimmed and quality filtered using cutadapt, simultaneously trimming low-quality bases from the 3′ end (−q 30) and removing reads below 50 bp in length (−m 50) [36]. Trimmed reads for each dataset were mapped to the appropriate reference genome (Table S1 [37–43]; using Bismark v0.17.0 [44] with adjusted mapping parameters (−-score_min L,0,-0.6). Reads from Dixon and Matz (2020) [18] were mapped using --non_directional mode as recommended by the Pico Methyl-Seq Library Prep Kit manual. PCR duplicates were removed from the Bismark alignment files using the deduplicate_bismark command. To estimate genomic coverage we computed the mean number of deduplicated reads across samples for each study, multiplied this value by the combined paired end read length, and divided by the summed length of the reference genome used. Methylation levels were extracted from the alignments using bismark_methylation_extractor with the --merge_non_CpG, −-comprehensive, and --cytosine_report arguments. Detailed steps used to process the WGBS reads are available on the git repository [45].

### RNA-seq data processing

Raw reads were trimmed and quality filtered using cutadapt, simultaneously trimming low-quality bases from the 3′ end (−q 30) and removing reads below 50 bp in length (−m 50, 36]. Trimmed reads for each dataset were mapped to the appropriate reference genome (Table S1) using Bowtie2 using the --local argument [46]. PCR duplicates were removed using MarkDuplicates from Picard Toolkit [47]. Sorting and conversion from sam files were performed using Samtools [48]. The reads mapping to annotated gene boundaries were counted using FeatureCounts [49]. Detailed steps used to process the RNA reads are available in the git repository [45].

### Measuring GBM level

Based on previous findings that different measures of GBM were highly similar [18], we reported GBM level as the percent methylation rate on the log_2_ scale. Here the percent methylation rate for a gene is the ratio of the total number of methylated read counts to read all counts summed across all CpG sites within the bounds of the gene. To allow plotting on the log scale, zero values were assigned to the lowest non-zero value for each project. Following previous studies [17, 18, 50], in the case of MBD-seq we report GBM as the log_2_ fold difference between the captured and unbound fractions generated during library preparation. GBM level based on mdRAD was computed as Reads per Kilobase of gene length per Million reads (RPKM) on the log_2_ scale. Analyses of differential methylation based on bisulfite sequencing data were done using MethylKit package [51]. Based on visual inspection of the distributions of methylation levels across genes in each species, we divided genes into highly methylated and lowly classes using a hard cutoff of 2.5% methylation.

### Relationships between GBM and mRNA

For each dataset, we tested for expected relationships between GBM and mRNA expression patterns. For our dataset, generated using Tag-seq [35], we calculated mean mRNA level by averaging the regularized counts generated using the rlog function in DESeq2 across all samples [52]. For the other datasets, which used standard RNA-seq, we calculated mean mRNA level as RPKM averaged across all samples. Differences in mRNA abundance between groups were calculated using DESeq2 [52]. For our dataset, this analysis was performed including colony identity (genotype) as a factor to control for genetic effects. For simplicity, models for differential expression for the published datasets included only the treatment groups indicated in Fig. 1 (we did not include additional factors, for instance, sex or colony identity). Differences between groups are reported as log_2_ fold differences. General transcriptional variation was estimated based on the coefficient of variation (standard deviation / mean) in Reads Per Kilobase Million Reads (RPKM) for the control samples from each study as in Huh et al. (2013) [53].

## Supplementary Information

**Additional file 1: Table S1**: Reference genomes used in this study.

**Additional file 2: Figure S1**: The coefficient of variation for transcription (standard deviation (RPKM) / mean(RPKM); computed from control replicates) was negatively related to GBM level (averaged across all samples).

**Additional file 3: Figure S2**: GBM and transcriptional differences between polyp types in *A. millepora* show no reproducible relationship. The title of each panel indicates the assay used to measure GBM differences. All axes are on the log_2_ scale.

**Additional file 4: Figure S3**: Significant differential GBM and differential transcription between phenotypic conditions show little or no relationship. The top set of panels shows the relationship between transcription and GBM differences for differentially expressed genes (DESeq2 FDR < 0.1). The bottom set of panels show the relationship for differentially methylated genes (MethylKit FDR < 0.1). All axes are on the log_2_ scale. The contrasts for each study are given in Fig. 1.

**Additional file 5: Figure S4**: Promoter methylation and transcriptional differences between polyp types in *A. millepora* show no relationship.

**Additional file 6: Figure S5**: Promoter methylation and transcriptional differences between phenotypic conditions show no relationship.

**Additional file 7: Figure S6**: GBM and transcriptional differences between experimental conditions show little or no relationship. Each pair of volcano plots is associated with the scatterplot below. The first volcano plot shows differences in GBM, with significant genes (q-value from MethylKit < 0.1) shown in red. The second shows differences in transcription, with significant genes in blue (FDR < 0.1). The count of significant genes is given above each volcano plot. The scatterplots are the same as those shown in Fig. 5, with expression differences on the Y axis and GBM differences on the X. The contrasts for each study are given in Fig. 1.

## Data Availability

The dataset(s) supporting the conclusions of this article are available in the NCBI SRA database, PRJNA415358, PRJNA533306, PRJNA437497, PRJNA304722, PRJNA598980, PRJNA598995, PRJNA386774, PRJNA104931, PRJNA309979. https://www.ncbi.nlm.nih.gov/sra. Intermediate datasets, code, and detailed data processing steps are available on github: https://github.com/grovesdixon/invert_meth_and_transcription.

## Abbreviations

GBM: Gene body methylation
RPKM: Reads Per Kilobase Million reads
WGBS: Whole Genome Bisulfite Sequencing
MBD-seq.: Methylation Binding Domain Sequencing
mdRAD: methylation dependent RAD-seq
