# Supplementary material for: Changes in gene body methylation do not correlate with changes in gene expression in Anthozoa or Hexapoda
Source: BMC Genomics. 2022 Mar 25;23:234. doi: 10.1186/s12864-022-08474-z (PMC8957121; doi:10.1186/s12864-022-08474-z)
Supplement: Supplementary file 1 — Additional file 1: Table S1: Reference genomes used in this study. [file 12864_2022_8474_MOESM1_ESM.docx]

Table S1: Reference genomes used in this study

| **Species** | **Bioproject** | **Accession** | **Reference** |
| --- | --- | --- | --- |
| *Ooceraea biroi* | PRJNA420369 | GCA_003672135.1 | (McKenzie and Kronauer 2018) |
| *Apis mellifera* | PRJNA471592 | GCA_003254395.2 | (Wallberg et al. |
| *Bombus terrestris* | PRJNA45869 | GCA_000214255.1 | (Sadd et al. 2015) |
| *Ceratina calcarata* | PRJNA299559 | GCA_001652005.1 | (Rehan et al. 2016) |
| *Zootermopsis nevadensis* | PRJNA396924 | GCA_000696155.1 | (Terrapon et al. 2014) |
| *Bombyx mori* | PRJDA20217 | GCF_000151625.1 | (Xia et al. 2008) |
| *Stylophora pistillata* | PRJDA20217 | GCA_002571385.1 | (Voolstra et al. 2017) |
| *Exaiptasia pallida* | PRJNA261862 | GCA_001417965.1 | (Baumgarten et al. 2015) |
| *Acropora millepora* | PRJNA633778 | GCA_013753865.1 | (Fuller et al. 2020) |
