# Supplementary figures and images for: Changes in gene body methylation do not correlate with changes in gene expression in Anthozoa or Hexapoda
Source: BMC Genomics. 2022 Mar 25;23:234. doi: 10.1186/s12864-022-08474-z (PMC8957121; doi:10.1186/s12864-022-08474-z)

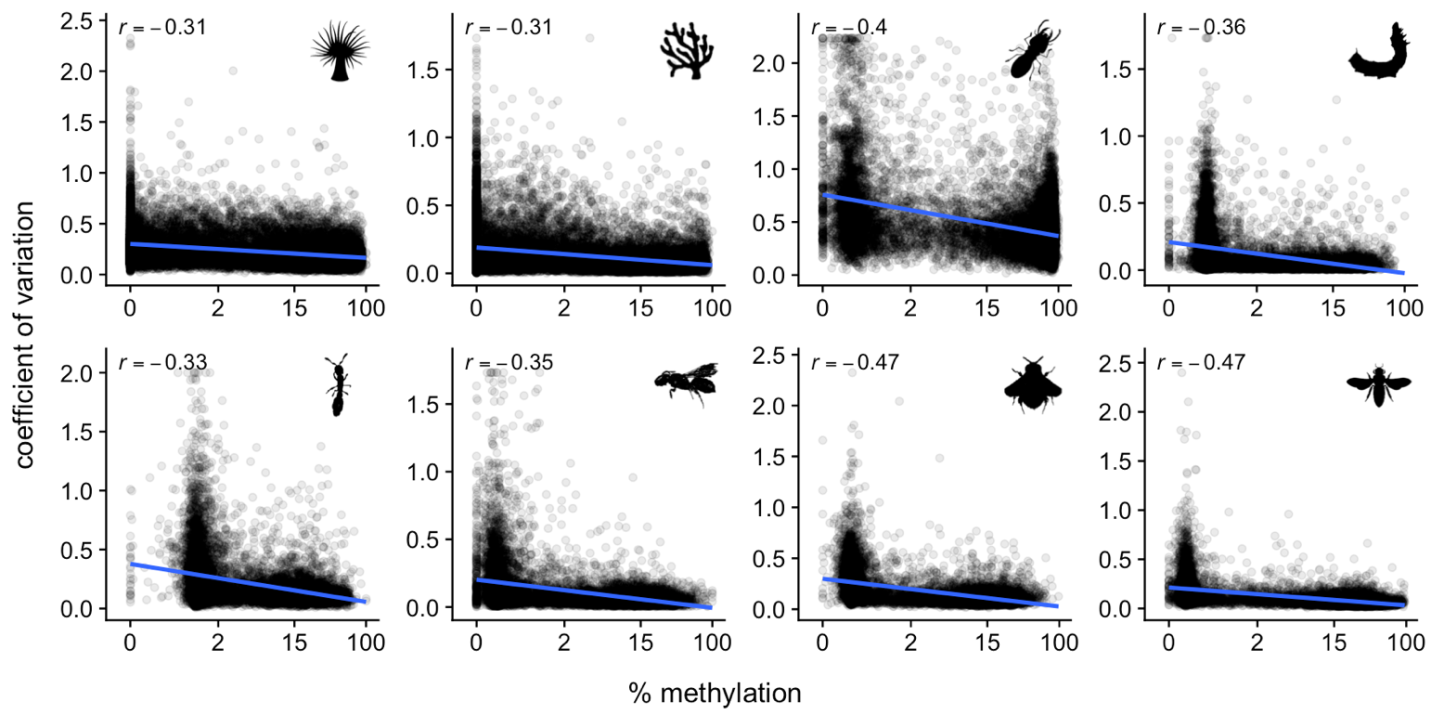

Supplement: Supplementary file 2 — Additional file 2: Figure S1: The coefficient of variation for transcription (standard deviation (RPKM) / mean(RPKM); computed from control replicates) was negatively related to GBM level (averaged across all samples). [file 12864_2022_8474_MOESM2_ESM.docx]

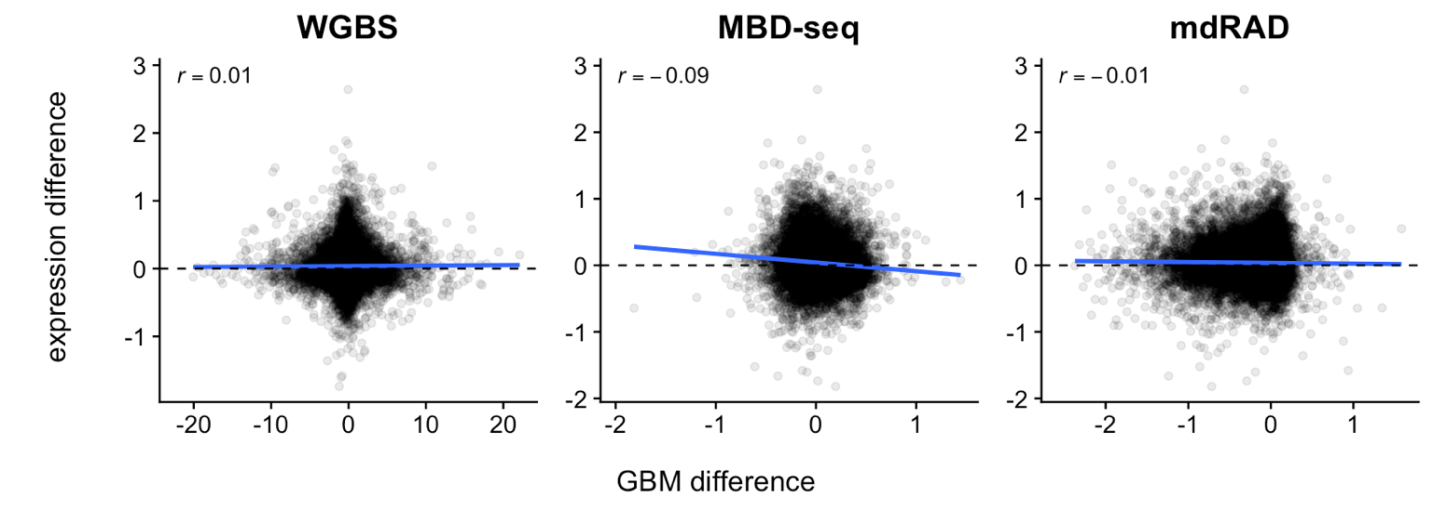

Supplement: Supplementary file 3 — Additional file 3: Figure S2: GBM and transcriptional differences between polyp types in A. millepora show no reproducible relationship. The title of each panel indicates the assay used to measure GBM differences. All axes are on the log2 scale. [file 12864_2022_8474_MOESM3_ESM.docx]

*
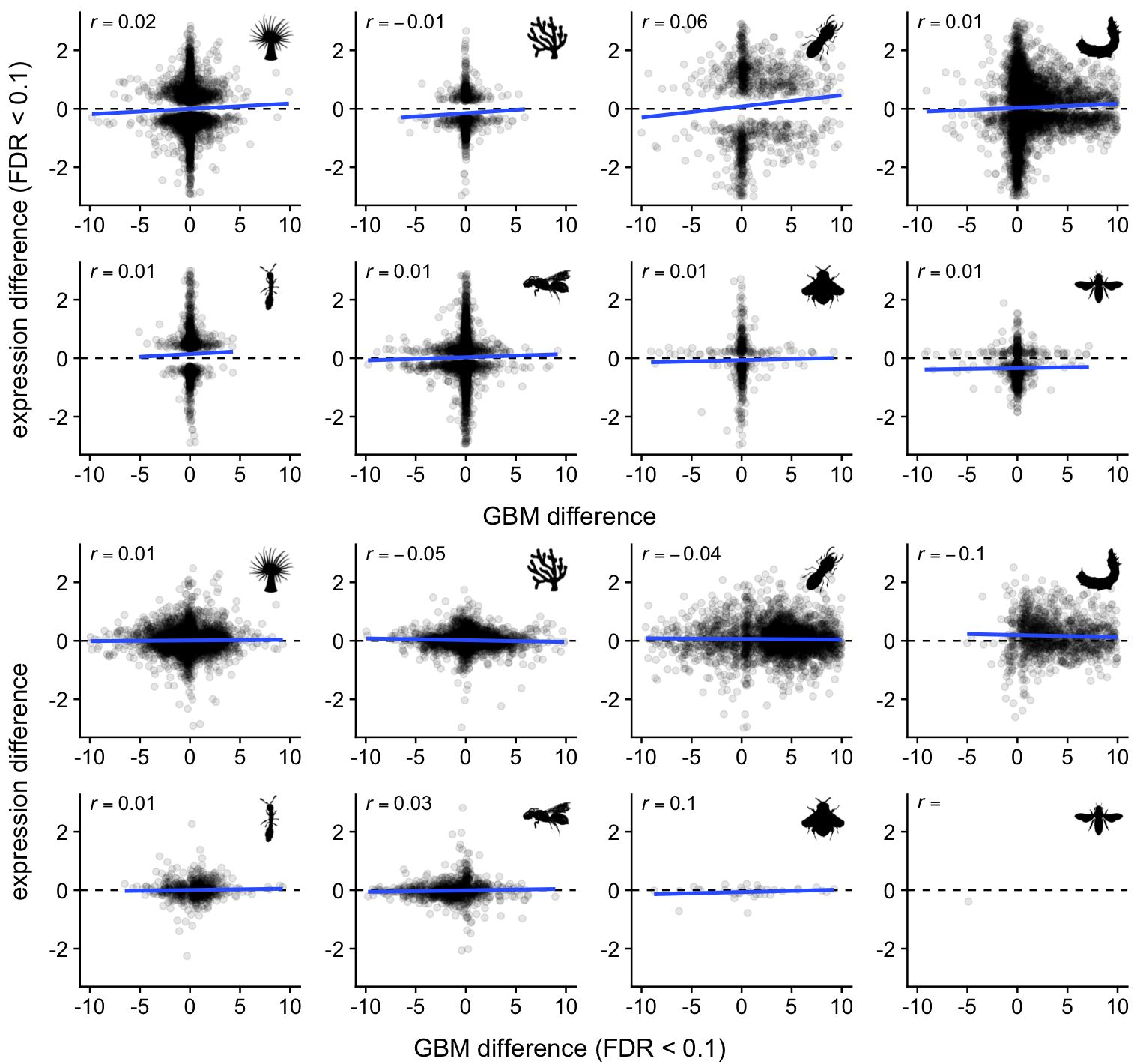
*

Supplement: Supplementary file 4 — Additional file 4: Figure S3: Significant differential GBM and differential transcription between phenotypic conditions show little or no relationship. The top set of panels shows the relationship between transcription and GBM differences for differentially expressed genes (DESeq2 FDR < 0.1). The bottom set of panels show the relationship for differentially methylated genes (MethylKit FDR < 0.1). All axes are on the log2 scale. The contrasts for each study are given in Fig. 1. [file 12864_2022_8474_MOESM4_ESM.docx]

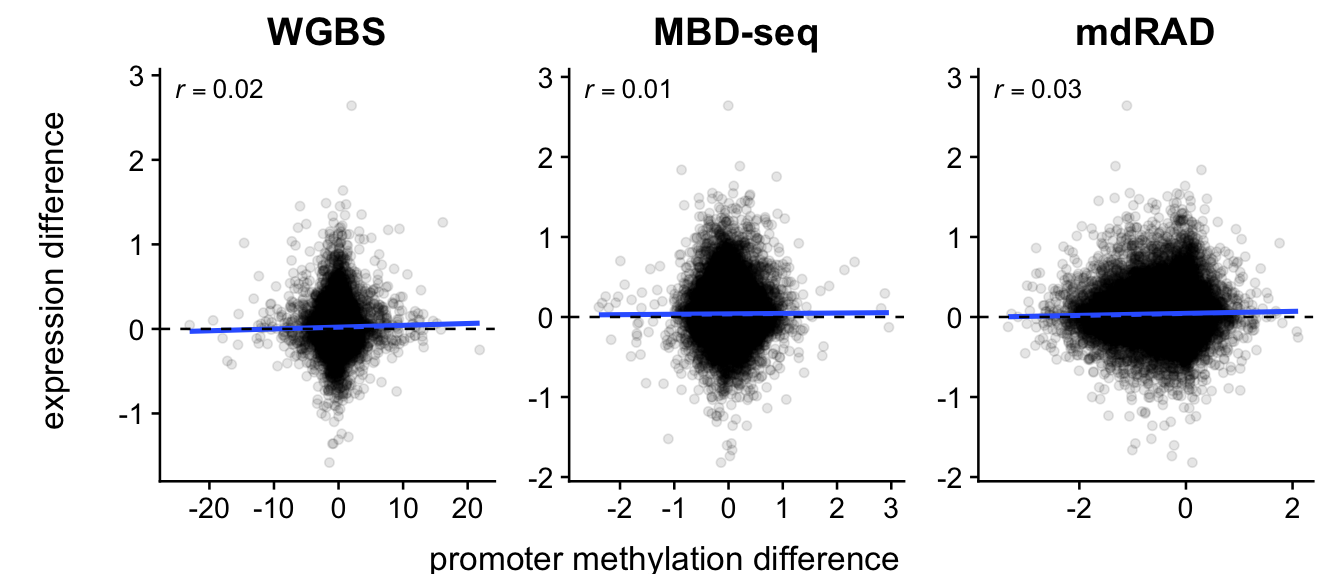

Supplement: Supplementary file 5 — Additional file 5: Figure S4: Promoter methylation and transcriptional differences between polyp types in A. millepora show no relationship. [file 12864_2022_8474_MOESM5_ESM.docx]

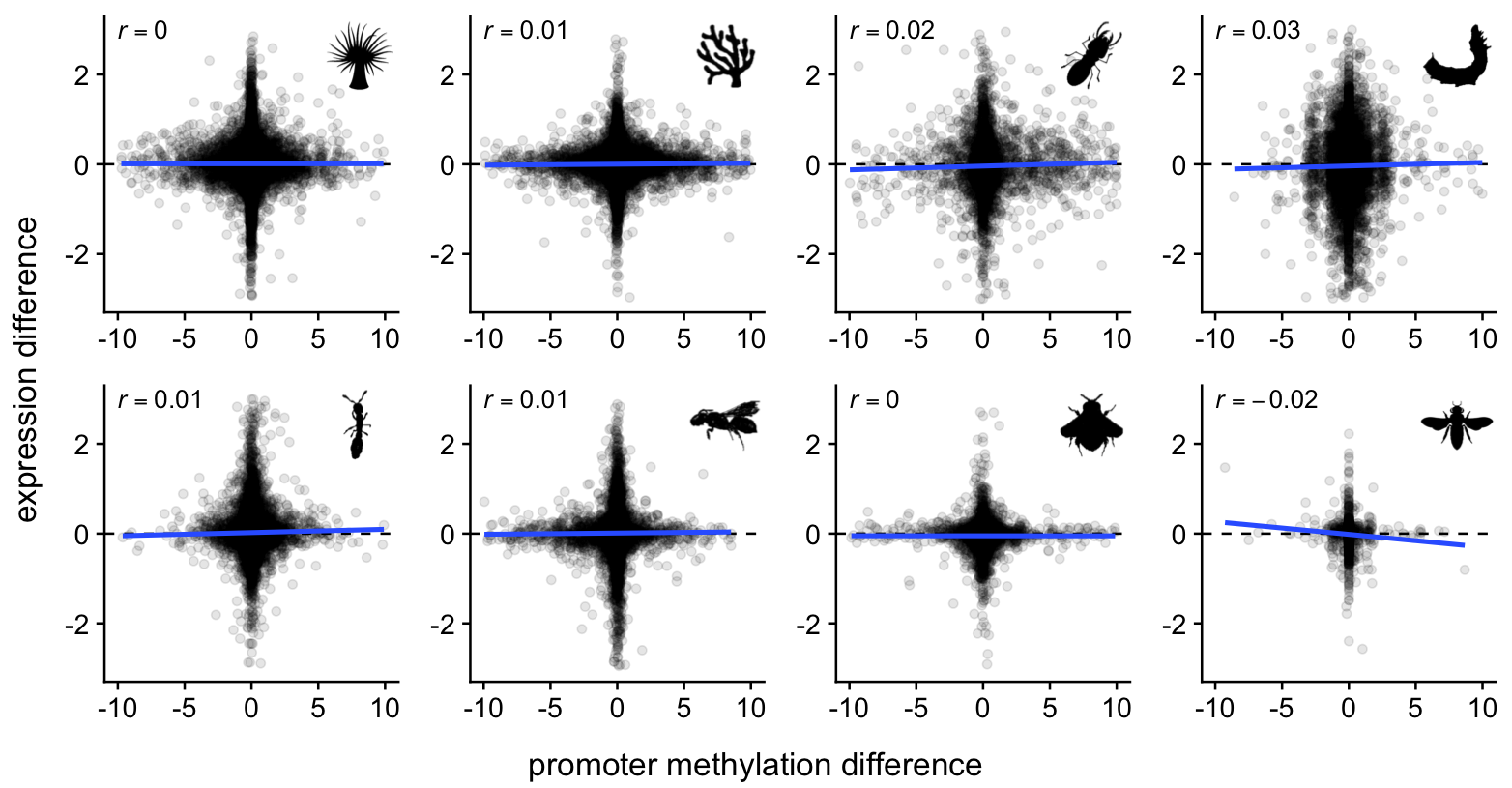

Supplement: Supplementary file 6 — Additional file 6: Figure S5: Promoter methylation and transcriptional differences between phenotypic conditions show no relationship. [file 12864_2022_8474_MOESM6_ESM.docx]

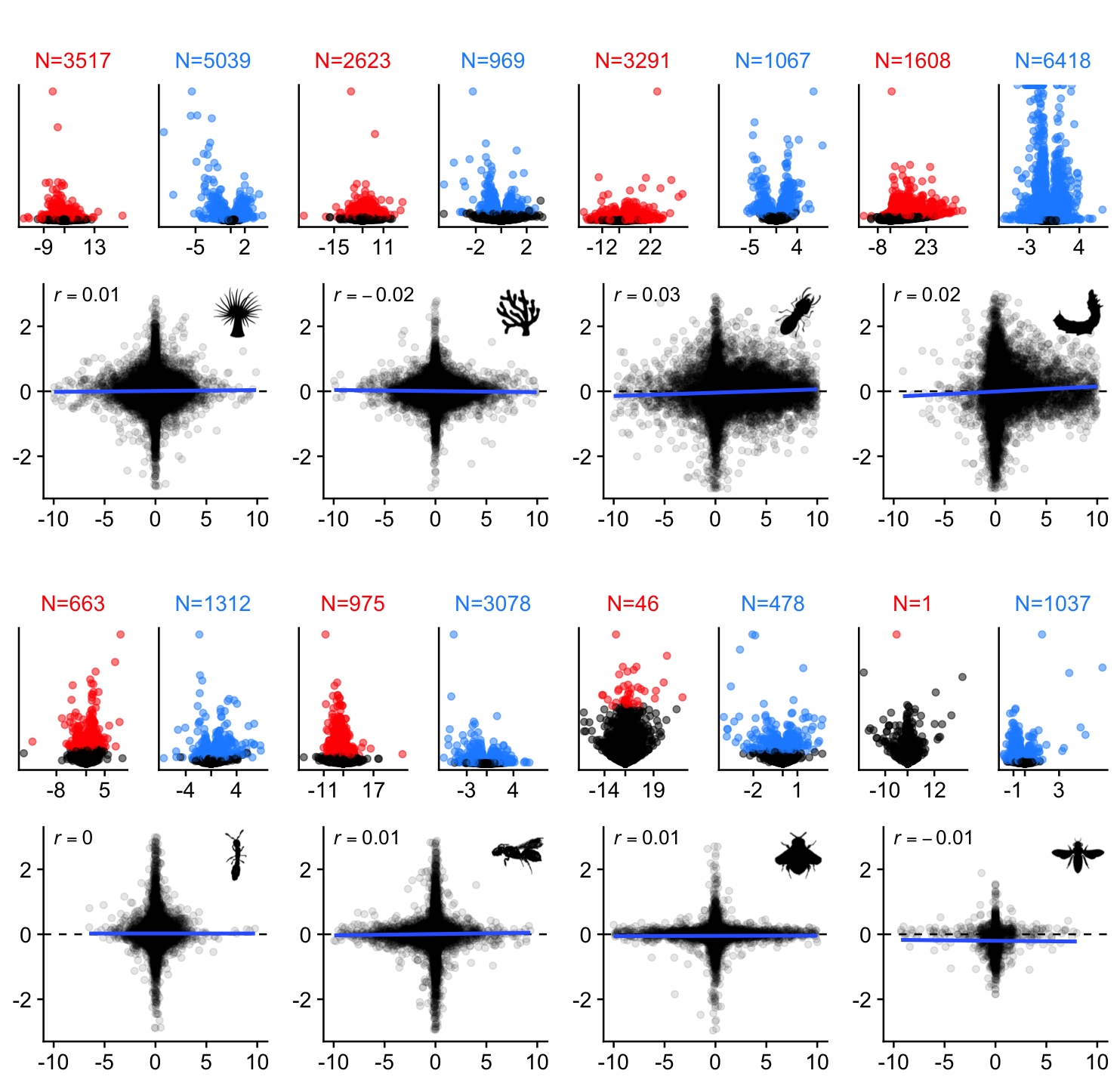

Supplement: Supplementary file 7 — Additional file 7: Figure S6: GBM and transcriptional differences between experimental conditions show little or no relationship. Each pair of volcano plots is associated with the scatterplot below. The first volcano plot shows differences in GBM, with significant genes (q-value from MethylKit < 0.1) shown in red. The second shows differences in transcription, with significant genes in blue (FDR < 0.1). The count of significant genes is given above each volcano plot. The scatterplots are the same as those shown in Fig. 5, with expression differences on the Y axis and GBM differences on the X. The contrasts for each study are given in Fig. 1. [file 12864_2022_8474_MOESM7_ESM.docx]
